# Identification and Characterization of microRNAs in the Developing Seed of Linseed Flax (*Linum usitatissimum* L.)

**DOI:** 10.3390/ijms21082708

**Published:** 2020-04-14

**Authors:** Tianbao Zhang, Zhen Li, Xiaxia Song, Lida Han, Limin Wang, Jianping Zhang, Yan Long, Xinwu Pei

**Affiliations:** 1 Biotechnology Research Institute, Chinese Academy of Agricultural Sciences, Beijing 100081, China; zhangtianbao@caas.cn (T.Z.); 18871433766@163.com (Z.L.); songxxia@163.com (X.S.); hanlida@caas.cn (L.H.); 2 Crop Institute, Gansu Academy of Agricultural Sciences, Lanzhou 730070, China; 13893414680f@163.com (L.W.); Z13038703697@163.com (J.Z.)

**Keywords:** microRNA, miRNA156, seed development, fatty acid synthesis, linseed flax

## Abstract

Seed development plays an important role during the life cycle of plants. Linseed flax is an oil crop and the seed is a key organ for fatty acids synthesis and storage. So it is important to understand the molecular mechanism of fatty acid biosynthesis during seed development. In this study, four small RNA libraries from early seeds at 5, 10, 20 and 30 days after flowering (DAF) were constructed and used for high-throughput sequencing to identify microRNAs (miRNAs). A total of 235 miRNAs including 114 known conserved miRNAs and 121 novel miRNAs were identified. The expression patterns of these miRNAs in the four libraries were investigated by bioinformatics and quantitative real-time polymerase chain reaction (qPCR) analysis. It was found that several miRNAs, including *Lus-miRNA156a* was significantly correlated with seed development process. In order to confirm the actual biological function of *Lus-miRNA156a*, over-expression vector was constructed and transformed to *Arabidopsis*. The phenotypes of homozygous transgenic lines showed decreasing of oil content and most of the fatty acid content in seeds as well as late flowering time. The results provided a clue that *miRNA156a* participating the fatty acid biosynthesis pathway and the detailed molecular mechanism of how it regulates the pathway needs to be further investigated.

## 1. Introduction

MiRNAs are non-coding RNAs about 20–25 nucleotide (nt) in length and they are encoded by endogenous genes, from which a primary non-protein coding message is transcribed (pri-miRNA) [1]. The pri-miRNA sequence contains an imperfect hairpin stem-loop structure allowing the molecule to fold-back onto itself to form dsRNA. Previous studies have confirmed that miRNAs regulate the expression of target genes through target mRNA degradation or translation inhibition [1,2,3]. As an important mechanism of post-transcriptional regulation, miRNAs could regulate genes involved in many developmental processes, such as flowering time, leaf development, auxin signaling and organ polarity [4,5,6,7,8].

MiRNAs are also found involving in the regulation of seed development process, such as *miR156*, *miR397*, *miR396* and *miR408*. For example, *miR396* regulates seed size and yield via its’ target gene *OsGRF4* in rice [9]. In lettuce, Huo et al. found that the suppression of *DELAY OF GERMINATION1* (*DOG1*) gene could enable seed germination at high temperature in associated with reduced *miR156* and increased *miR172* levels [10]. Seeds are important storage organs. For oil crops, several fatty acids like oleic acid, linoleic acid and linolenic acid are storage in the seeds. Until now, several miRNAs have been discovered via high-throughput sequencing during the seed development in different oil crops, such as soybean, rapeseed. In soybean, 55 annotated miRNAs and 26 new soybean miRNAs were detected in a seed small RNA library [11]. Using high-throughput technology, there are 85 known miRNAs from 30 miRNA families as well as 1610 novel miRNA at stages of different seed development in *B. napus* [12]. Among the abundant miRNAs, some specific RNAs have been found relating with fatty acid biosynthesis. For example, *bBna-miR156b*, *bna-miR156g*, *bna-miR159*, *bna-miR395b*, *bna-miR6029* and 19 novel miRNAs were found to be involved in fatty acid biosynthesis [12]. As a conservative miRNA existing in different crops, *miRNA156* was found to be involved in different aspects of agronomic traits, such as fruit development in tomato, tuberization in potato, nodulation in soybean [13]. For example, *miR156* could play important roles in the modulation of grape berry development and ripening [14,15]. *Vv-miR156* exhibited an overall increasing expression trend during berry development and ripening in grape [15]. Previous studies have confirmed that *miR156* regulating developing processed through the *SPL* gene family [16]. In *Arabdopsis thaliana*, *miR156* has ten targets, such as *SPL2*, *SPL3*, *SPL4*, *SPL5*, *SPL6*, *SPL9*, *SPL10*, *SPL11*, *SPL13* and *SPL15* genes [16]. *MiR156* targets *SPL10* and *SPL11* genes, which can cause abnormal cell division and control the development of seeds in *Arabidopsis thaliana* [17]. There were too many researches about *miR156* but researches involving in fatty acids metabolism of *miR156* are limited.

Flax is an annual herbaceous dicotyledonous plant, which is divided into oil flax, fiber flax and oil fiber dual-purpose flax [18]. Flax seed is not only a reproductive organ to maintain generation continuity but also an organ to store oil and its storage capacity directly affects oil content and grain yield. Linseed oil is rich in a variety of unsaturated fatty acids, such as oleic acid, linoleic acid, linolenic acid and so forth, especially the higher content of α-linolenic acid, the average content of 40% to 60%. To identify the key genes involved in fatty acids biosynthesis, a cDNA library made from flax bolls collected at 12 days after anthesis was constructed and screened for ketoacyl CoA synthase, fatty acid elongase, stearoyl-ACP desaturase and fatty acid desaturase [19].

In recent years, some progress has been made in the identification and functional analysis of miRNA in flax. Neutelings identified 20 conserved miRNAs belonging to 13 families [20]. In the following years, miRNAs that play a role in the absorption and reaction of nutrients, such as N and P, were identified in flax [21,22]. For example, Melnikova identified a total of 96 conserved miRNAs under normal and deficient phosphorus conditions and found 475 new potential miRNAs [21]. However, the role of miRNAs in flax seed development remains unclear. Therefore, the identification of miRNAs in linseed flax seed development and the clarification of their functions will help to understand the regulatory process of flax seed development.

## 2. Results

### 2.1. Small RNA Libraries Data Analysis

Four small RNA libraries, M5, M10, M20 and M30, from four development stages of flax seed were constructed. After high-throughput sequencing, 22,179,284, 34,145,577, 19,947,423 and 18,881,760 reads were successively obtained respectively for the four libraries.

Reads without 3’ adaptor sequence and insert fragment were removed. Sequences shorter than 18 or longer than 30 nucleotides were removed. 20,601,320, 13,039,680, 17,501,654 and 13,870,666 reads were successively obtained (Table 1). Using Bowtie software, clean reads were screened against Silva database, GtRNAdb database, Rfam database and Repbase database. NcRNAs including ribosomal RNA (rRNA), transfer RNA (tRNA), small nuclear RNA (snRNA), small nuclear RNA (snoRNA) and repeat sequences were filtered to obtain unannotated reads containing miRNA. The average ratio of the content of rRNA in four libraries was less than 20%, indicating that the quality of the four libraries constructed was reliable. The unannotated reads was 91.8%, 75.69%, 82.4% and 52.67%, respectively (Table 1). Then, the unannotated reads were compared to the flax genome and 11,568,108, 6,180,914, 9,606,035, 4,181,608 reads were matched to the flax genome, which accounted for 61.17%, 62.64%, 66.62% and 57.24% of the unannotated reads (Table 1).

### 2.2. Identification of Known and Novel miRNAs

**Figure 1:**
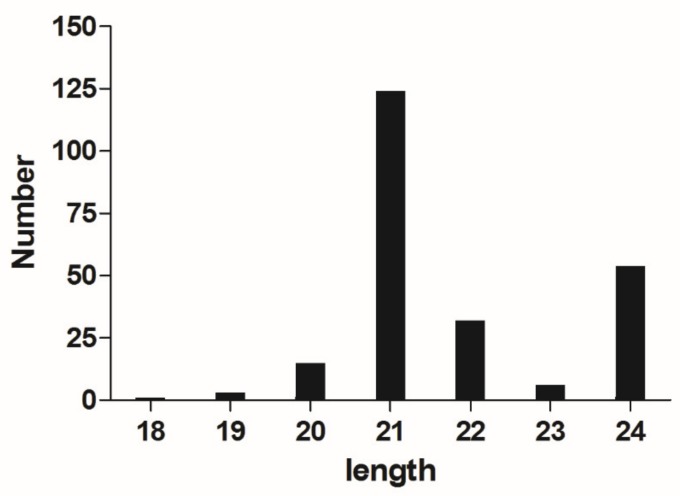
The length of miRNAs in the four libraries.

After screening the miRNAs, it was found that the length of the miRNAs varied from 18 nt-24 nt and the miRNA content of 21 nt was the highest, indicating that miRNAs with 21 nt played important roles in linseed flax seed development (Figure 1). MiRDeep2 software was used to identify known and new miRNAs. Through the alignment of reads to the flax genome, possible precursor sequences are obtained. Based on the distribution information of reads on the precursor sequences and the energy information of the precursor structures, the Bayesian model was used to score and finally realize the identification of miRNAs. Totally, there were 235 miRNAs predicted for all samples, including 114 known miRNAs and 121 newly predicted miRNAs (Table 2). For the 114 known unique miRNAs, there were 23 families, including the conserved miRNA156, *miRNA166*, *miRNA169* and et al. The family members ranged from one to 11. The *miR166* family was the largest family which having 11 members, followed by *miR156*, *miR167*, *miR169*, *miR171* and *miR172* with 9 members. The 121 newly predicted miRNAs distributed in 89 scaffolds and 5 contigs of the flax genome. The pri-sequences and mature sequences of all of the 235 miRNAs were listed in Supplementary Table S1.

### 2.3. Analysis of miRNA Expression in Four Developing Stages of Seeds

**Figure 2:**
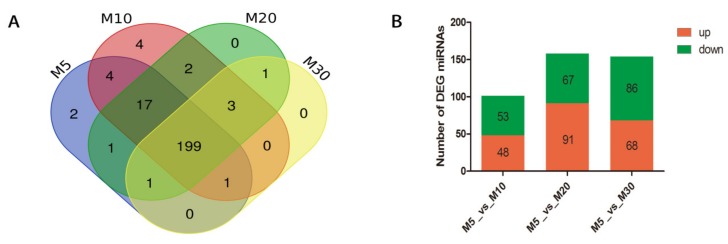
Comparison of the expression of miRNAs in the four libraries. (**A**). The numbers of miRNAs expressing in each of the library. (**B**). Differentially expressed miRNAs during seed development. M5 library was as a control.

After getting the miRNA sequences, the expression values of all the miRNAs were calculated. Among all the 235 identified miRNAs, 199 miRNAs co-expressed in the four stages of seed development. Two miRNAs, *lus-miR169g* and *lus-miR169l*, specifically expressed in M5 library and four miRNAs (*lus-miR156d*, *lus-miR171a*, *lus-miR171f* and *lus-miR828a*) specifically expressed in the M10 library (Supplementary Table S2). There were no miRNAs specifically expressed in M20 and M30 libraries. There were 221 miRNAs co-expressed in M5 and M10 libraries, while 4 and 9 expressed separately in M5 and M10 libraries. 118 miRNAs co-expressed in M5 and M20 libraries while 7 and 6 expressed separately in M5 and M20 libraries. There were 24 and 4 expressed separately in M5 and M30 libraries, while 201 miRNAs co-expressed in M5 and M30 (Figure 2, Supplementary Table S2).

Then, the expression values of miRNAs in each sample were statistically analyzed and normalized by TPM algorithm. |log2(FC)| ≥ 1 and FDR ≤ 0.01 were used as screening criteria in the detection of differentially expressed miRNAs. After calculating the expression values of the miRNAs, 101, 158 and 154 miRNAs showed significantly different expression between 5 DAF and 10 DAF, 5 DAF and 20 DAF and 5 DAF and 30 DAF libraries, respectively. For 101 differentially expressed miRNAs were detected between M10 and M5 libraries, 48 were up-regulated and 53 were down-regulated. There were 158 differentially expressed miRNAs in M20 and M5 libraries, among which 91 were up-regulated and 67 were down-regulated. A total of 154 differentially expressed miRNAs were detected in M30 and M5 libraries, of which 68 were up-regulated and 86 down-regulated (Figure 2).

### 2.4. Target Gene Prediction and GO Analysis for the Differentially Expressed Target Genes

In general, miRNA and target genes are generally negatively regulated. In order to find target genes of the identified miRNAs, TargetFinder software was used to predict. As a result, for the 235 identified miRNAs, only 109 miRNAs have been predicted 630 targets (Supplementary Table S3). For the 114 known conserved miRNAs, 85 miRNAs had predicted target genes, while for the novel miRNAs, only 24 miRNAs had predicted target genes. Many known miRNA families, such as *lus-miRNA156* (*a*–*i*), *lus-miRNA160* (*a*–*j*), had different potential functional target genes, which mean that these miRNAs are involved in regulating multiple genes’ expression in linseed flax.

**Figure 3:**
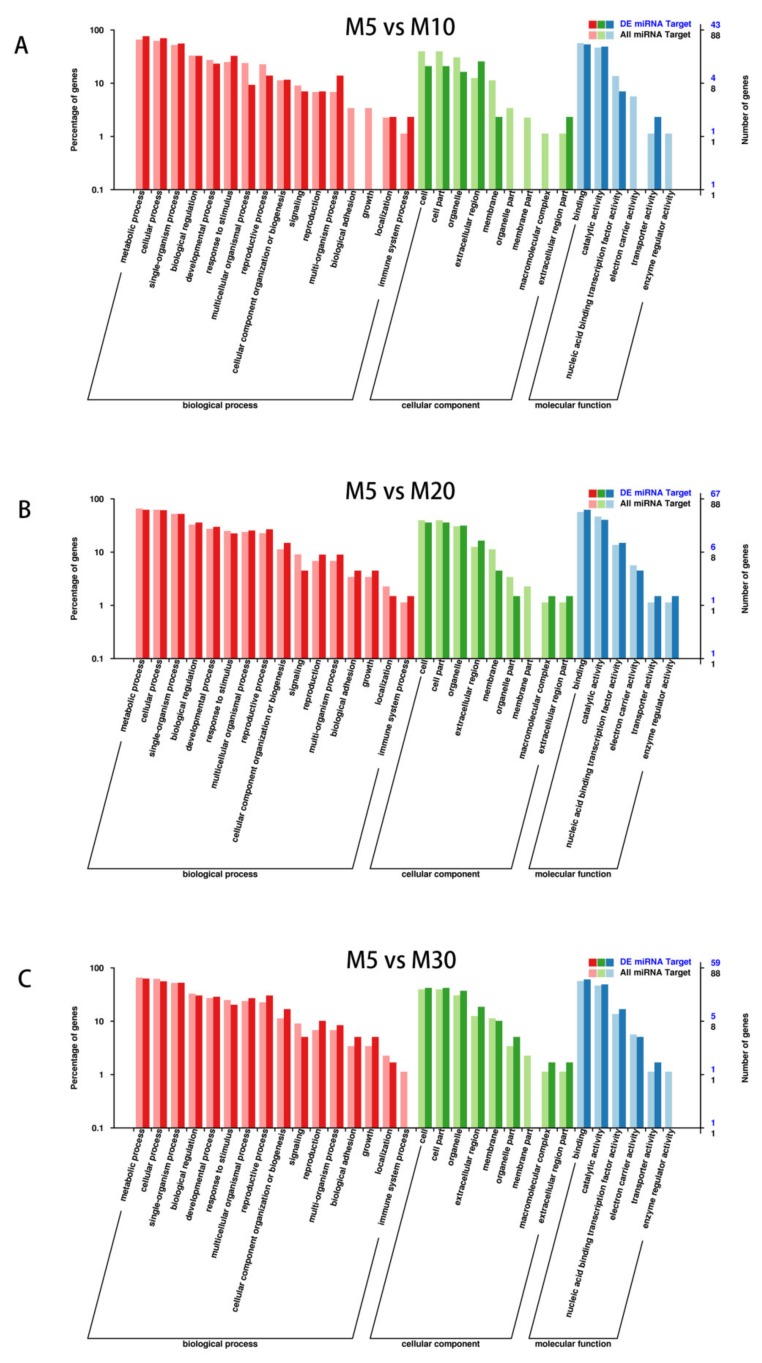
Gene ontology classifications of miRNA targets and differentially expressed targets in seed development. (**A**) M5 vs. M10, (**B**) M5 vs. M20, (**C**) M5 vs. M30.

Comparing the M5 and M10 libraries, there were 43 significantly altered genes. There were 67 significantly altered genes between M5 and M20 libraries. Meanwhile, 59 significantly altered genes between M5 and M30 libraries. Then GO analysis including three major types was used to classify the gene function of the target and differentially expressed target genes. For the targets, there were 6 molecular functions, 16 biological processes and 9 cellular components were included. For differentially expressed target genes between M5 vs. M10 library, 4 molecular functions, 14 biological processes and 6 cellular components were included. For M5 vs. M20 library, 6 molecular functions, 16 biological processes and 8 cellular components were included. And for M5 vs. M30 library, the differentially expressed genes could be divided into 5 molecular functions, 15 biological processes and 8 cellular components (Figure 3). Several biological processes including metabolic process, biological regulation and cellular process were included.

### 2.5. MiRNA Expression Verification by Using qRT-PCR 

**Figure 4:**
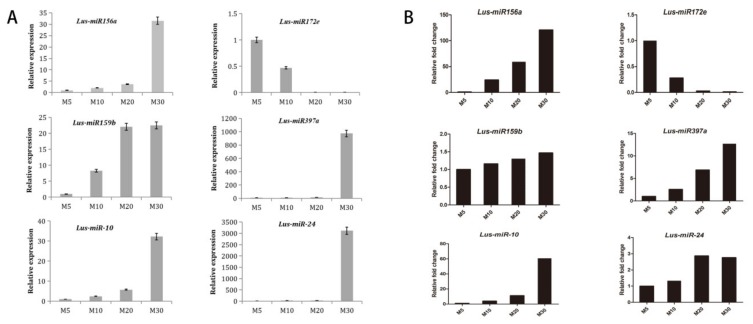
Expression patterns of *miR156a*, *miR172e*, *miR159b*, *miR397a*, *Lus-miR-10* and *Lus-miR-24* in seed developmental stages (M5, M10, M20 and M30) of qPCR and Next-generation sequencing (NGS) data. Error bars indicated standard deviation of three replicates. (**A**)The results of qPCR, (**B**) The results from NGS data.

To confirm the sequencing results and examine the expression patterns of the miRNAs at different stages of seed development (M5, M10, M20 and M30), four known conserved miRNAs including *Lus-miR156a*, *Lus-miR172e*, *Lus-miR159b*, *Lus-miR397a* and two novel identified miRNAs, *Lus-miR-10* and *Lus-miR-24* were used to perform qPCR (Figure 4). As the seed develops to maturity, *Lus-miR156a*, *Lus-miR159b* and *Lus-miR-10* was steadily increasing from M5 to M30 stage. For *Lus-miR397a* and *Lus-miR-24,* they expressed very low in the 5 DAF to 20 DAF, while the expression values increased much in the 30 DAF. And *Lus-miR172e* was down-regulated during the seed development process. The expression tendency was same after comparing with the high-throughput sequencing result (Figure 4). The results showed that the sequencing results of the four libraries in this study was reliable and the identified miRNAs could be further investigated for illustrating the relationships between them and seed development and even fatty acid synthesis. 

### 2.6. Screening of Target Genes of Lus-miR156a in Flax Genome

**Figure 5:**
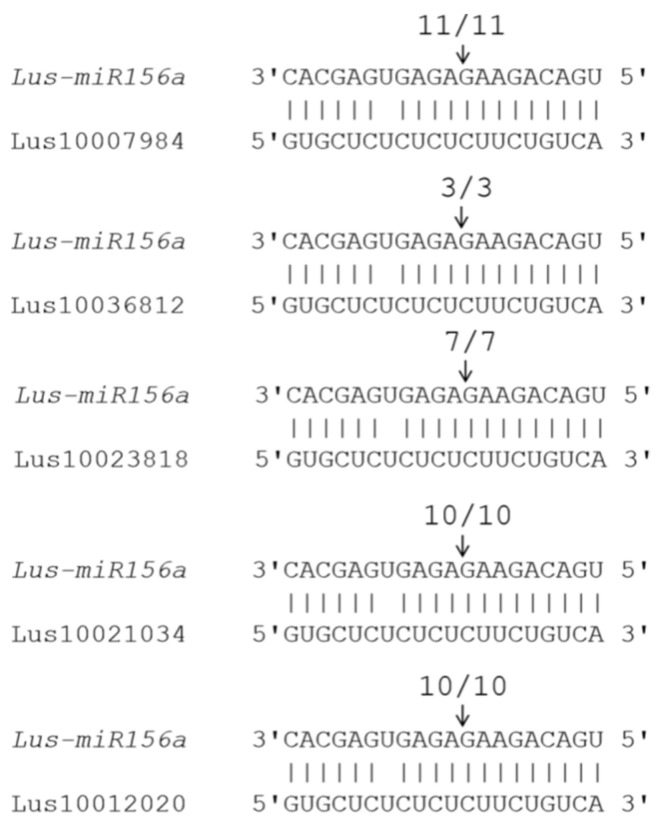
Mapping target mRNA cleavage sites by 5’-RLM-RACE. The arrows indicate the cleavage sites and the numbers show the frequency of clones sequenced.

Based on the miRNA identification and differentially expressed gene analysis, it was inferred that *Lus-miR156a* and its’ related target genes could participate the seed development process. So then the cleavage sites of target genes were screened by using the 5’-RLM-RACE method, so as to determine the real target gene of miRNA156. The results were shown in Figure 5. It showed that totally five target genes of *lus-miR156a* having cleavage sites, which are cleaved between the 11th and 12th bases in the complementary region. After searching the potential gene functions of these five genes, it was found that they were homology with the *SPL* genes, especially had highly similarity with *SPL6* and *SPL9* genes in *Arabidopsis* genome.

### 2.7. Overexpression of miR156a Affects the Flowering Time, Rosette Leaves and Fatty Acids

**Figure 6:**
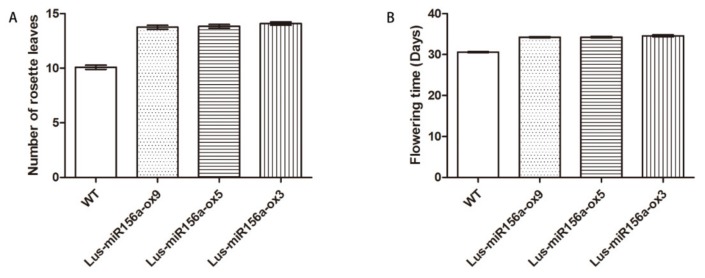
Number of rosette leaves and flowering time of the transgenic lines and wild type. (**A**) Number of rosette leaves, (**B**) Flowering time.

To analyze the function of *Lus-miR156a*, the overexpression plasmid vector was constructed and transformed it to *Arabidopsis*. Then the phenotypes of flowering time, rosette leaves and fatty acids of the homologous transgenic lines of T3 generation were investigated. For the rosette leaves of *Lus-miR156a* overexpression line were 3.67–4.00 leaves than *Arabidopsis* wild-type (Figure 6A). And for flowering time, it was found that the flowering time of *Lus-miR156a* overexpression lines delayed 3.67–4.00 day than *Arabidopsis* wild-type (Figure 6B). After getting the mature seeds, the fatty acid content and oil content were analyzed by GC-MS. The total oil content of the transgenic lines decreased 10% compared to that of the wild type (Table 3). Besides the total oil content, the fatty acid contents of the seed were investigated. It was found that the levels of C16:0, C18:0, C18:2^Δ9^^,12^, C18:3^Δ9,12,15^, C20:2^Δ11^^,14^ in *Lus-miR156a* overexpression lines were significantly lower than that of the wild type. It means that *Lus-miR156a* actually participate the fatty acid metabolism pathway.

### 2.8. Seed Oil Synthesis Genes Were Regulated by Lu-miR156a in Arabidopsis Transgenic Lines

**Figure 7:**
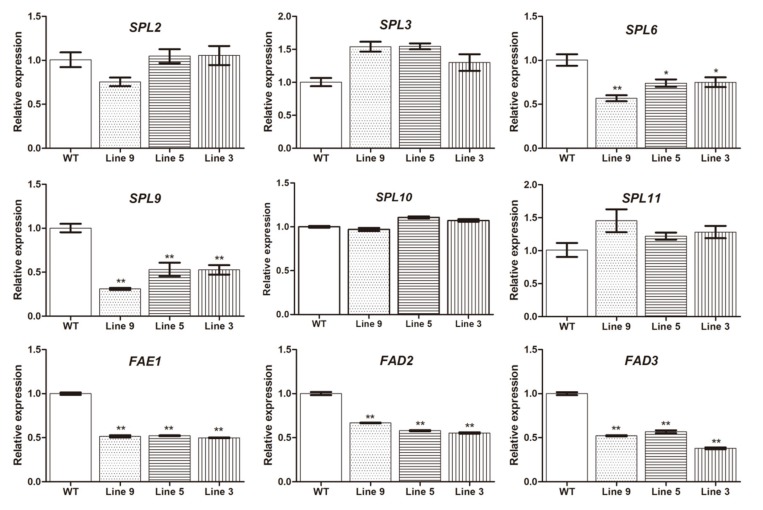
Expression levels of SPL genes and seed oil synthesis genes in the over-expression *Arabidopisis* transgenic lines and WT. Line 9: Lus-MIR156a-OX9, Line 5: Lus-MIR156a-OX5, Line 3: Lus-MIR156a-OX3. * *p* < 0.05 and ** *p* < 0.01 indicated significant differences with WT.

In the *Lu-miR156a* over-expression transgenic lines, the target gene, including *SPL6* and *SPL9*, were significantly decreased than WT, while the other *SPL* genes, such as *SPL3*, *SPL10, SPL11* were not significantly changed compared to WT (Figure 7). This result showed that *SPL6* and *SPL9* genes were target genes of miR156a in seed development process. In order to evaluate how the seed oil synthesis genes regulated by *Lu-miR156a*, some oil synthesis related genes were used. The results showed that *FAD2*, *FAD3* and *FAE1* were significantly decreased in transgenic lines (Figure 7). It mean that over-expression of *Lu-miRNA156a* influenced the gene expression of seed oil synthesis genes.

## 3. Discussion

As an important variety of oil crops, there are some studies involving in miRNAs for linseed flax, while until now there are few studies focusing on seed development or fatty acid synthesis. In this study, 235 miRNAs were predicted based on four libraries from four seed developing stages, which including 114 known miRNAs belonged to 23 families and 121 novel miRNAs. Then, a conservative miRNA, *lus-miRNA156a* was further investigated for its’ target genes and potential biological functions. The phenotypes of transgenic lines showed *lus-miRNA156a* could regulate flowering time. The most interesting result of this study was that over expression of *Lus-miRNA156a* could change the fatty acid content and total amount of the oil content in seeds. This result provided the new evidence that miRNA156a could attend regulating the fatty acid synthesis pathway in linseed flax.

In this study, four small RNA libraries, M5, M10, M20 and M30, were constructed for sequencing. Totally 235 miRNAs were identified correlating with the seed developing process. Compared the miRNA numbers to the previous published researches, it was found that the miRNA numbers would increase when the libraries increased. In soybean, a small RNA library, 15 DAF, was used for miRNA identification and totally 207 miRNAs were identified [11]. There were also 85 unique miRNAs identified when using three small RNA libraries in *B.napus* [12]. This mean that more samples will help to identify more miRNAs which regulate specific developing process in plants, including seed development. Among the 235 miRNAs, the 114 unique miRNAs belonged to 23 families. With the 23 families, the *miR166* family was the largest family which having 11 members, followed by *miR156*, *miR167*, *miR169*, *miR171* and *miR172* with 9 members. The tendencies of the results were similar with previous studies and with some differences. Wang et al. found that the *miR169* family was the largest family with 10 members in the three small miRNA libraries of *B.napus* seeds [12]. While also in *B.napus* seeds, Korbes et al. found *miR156*/*157* was the largest family (24 members), followed by the *miR165*/*166* (21 members) and *miR169* (15 members) families. There were 2–6 members were found existing in the remaining miRNA families identified [23]. The different expression of these small RNAs in different oil corps suggest that they function in common and unique regulation pathways.

Since the miRNA and target duplexes are near-perfectly matched in plants, it is possible to find targets by computational approach. In the current study, for the 235 identified miRNAs, 109 miRNAs have 630 target genes. The 85 conserved miRNAs had target genes, while only 24 novel miRNAs had target genes, which meant that conserved miRNAs had more target genes than that of the novel discovered miRNAs. The reason is that conserved miRNAs play key roles in universal mechanisms of regulation in different plant varieties. While the linseed flax specific miRNAs may only function in regulation of gene expression during flax seed developing stages. Meanwhile, most of the target genes of conserved miRNAs are transcription factors and the linseed flax specific miRNAs may regulate various types of genes. It means that there is a new feature of miRNA regulation pathway in linseed flax.

In general, the miRNAs act to down-regulate their target genes by directing cleavage of the highly complementary target transcripts. In *Arabidopsis*, of the 16 Squamosa-promoter Binding Protein (SBP)-like genes, ten have *miR156* complementary sites [24,25]. 19 rice *SPL* genes and 12 rice *miRNA156* precursors were identified in the rice genome. Sequence and experimental analysis suggested that 11 *OsSPL* genes were putative targets of *OsmiR156* [26]. Ten *SlySBP* genes carry putative miR156-binding sites in tomato [27]. Using the 5’-RLM-RACE method, we found the five target genes of *miR156a* have cleavage sites, whose cleaved site is between the 11th and 12th bases in the complementary region. BLAST analysis showed that these five target genes were closet similarity with *SPL6* and *SPL9* of *Arabdopsis*. *SPL9* have a major function in both the vegetative-to-reproductive transition and the juvenile-to-adult vegetative transition [16]. *SPL6* does not contribute to in shoot morphogenesis but may be important for certain physiological processes [16]. The gene expressions of *SPL6* and *SPL9* were significantly down-regulated in *Lu-miR156a* over-expression plants, while the other four *SPL* genes were not regulated. It mean that *SPL6* and *SPL9* were target genes of *miR156a* in seed developing process. The data suggested that different crops contain miRNA with different target genes in different organs.

MiRNAs play important roles during plant growth, developmental transitions and determination of cell identity [28,29]. Seed is a crucial organ of plant. There are many researches on the development of seed development. While for miRNAs, most of the researches are based on high-throughput sequencing. For example, in oil seed crops, such as soybean, rapeseed, many miRNAs have been discovered for correlating with the seed development. While until now, there were no direct clues which could confirm the regulating pathway between miRNAs and seed development or fatty acid synthesis. The current study provided a new clue for *miRNA156* regulating the fatty acid metabolism. Previous studies have shown that *miR156* regulates many different aspects of developing. For flowering time, overexpression of *miR156* in both *Arabidopsis* and maize prolongs the expression of juvenile vegetative traits and delays flowering [30,31]. Also in our study, *miR156a* can increase the number of rosettes leaves, leading to delay the flowering time. Besides the flowering time and rosette leaves phenotypes, the fatty acids profiles of overexpression *miR156a* lines were changed, the total oil content of the transgenic lines decreased comparing with that in wild type, also the fatty acid content in the seeds. It indicates that *Lus-miR156a* plays a certain role in fatty acid metabolism. For the specific fatty acid, the linoleic acid, the linolenic acid contents were decreased. The three genes, *FAD2*, *FAD3* and *FAE1*, which mainly regulated the synthesis of these two fatty acids, were down-regulated in the transgenic plants compared to WT. It mean that miRNA156a actually regulated the gene expression of seed oil synthesis pathway. While the actual regulating mechanism of how miRNA156a work on seed oil synthesis and metabolism through regulating its’ target genes need to be further investigated. 

## 4. Materials and Methods

### 4.1. Plant Materials and Growth Condition

One linseed flax cultivar, Macbeth, was used as material for miRNA identification. Its oil content is about 46.7% and linolenic content is about 44.5% [32]. The cultivar was planted in the field trial station of Chinese Academy of Agricultural Sciences in Langfang city, Hebei province, China. The blooming flower was tagged and then the siliques were collected respectively in 5, 10, 20 and 30 days after flowering. All the samples were put in liquid nitrogen immediately for later RNA extraction.

### 4.2. RNA Extraction and Small RNA Library Construction

Total RNA was extracted using the EASY spin Plant microRNA Kit (Aidlab, Beijing, China). NEB Next Ultra small RNA Sample Library Prep Kit for Illumina (NEB, Ipswich, MA, USA) was used for small RNA Library construction. Sequencing was performed using Illumina HiSeq2500 high-throughput sequencing platform (Biomarker Technology, Beijing, China).

### 4.3. Analysis of Sequencing Results

The sequencing raw reads with the content of unknown base N greater than or equal to 10% were removed. Reads without 3 ’adaptor sequence and inserted fragment were removed. Sequences shorter than 18 nucleotides or longer than 30 nucleotides were removed and clean reads were obtained. Using Bowtie software, clean reads respectively with Silva database(http://www.arb-silva.de/), GtRNAdb database(http://lowelab.ucsc.edu/GtRNAdb/), Rfam database(http://rfam.xfam.org/) and Repbase database(http://www.girinst.org/repbase/) sequence alignment. To obtain unannotated reads containing miRNA, ribosomal RNA (rRNA), transfer RNA (tRNA), small nuclear RNA (snRNA), small nuclear RNA (snoRNA) and repeat sequences were filtered. Clean reads were compared with the flax genome (https://phytozome.jgi.doe.gov/pz/portal.html#!info?alias=Org_Lusitatissimum) using the Bowtie software and the location information on the reference genome was obtained, that is, mapped reads. Identification of known and new miRNAs were performed with miRDeep2 software using the criteria for plant miRNAs annotation [33]. According to the known miRNA and newly predicted miRNA and flax gene sequence information, TargetFinder (https://www.acunetix.com/blog/docs/target-finder/) was used for target gene prediction.

### 4.4. Expression of miRNAs

High-throughput sequencing data was normalized the expression levels with TPM algorithm [34]. |log2(FC)|≥1 and FDR≤0.01 were used as screening criteria in the detection of differentially expressed miRNA. qPCR was then used to verify miRNAs expression. Ten ng RNA was reversed using TaqManH miRNAs Reverse Transcription Kit (Applied Biosystems, USA). The obtained cDNA was amplified using a miRNA Universal SYBR qPCR Master Mix (Vazyme, Nanjing, China) Kit in ABI7500 real-time System (Applied Biosystems, Foster, CA, USA) with actin as internal reference. The 2^−ΔΔ*C*t^ method expression levels of miRNA in different materials were calculated using the comparative Ct method [35,36]. Four known miRNAs, such as *miR156a*, *miR159b*, *miR172e* and *miR397a* and two new miRNAs, such as *Lus-miR-10* and *Lus-miR-24*, were performed qPCR to analyze their expression level.

### 4.5. GO Analysis for Target Genes and Differentially Expressed Genes

In order to understand the targets of miRNAs and classifications as well as the metabolic regulatory networks associated with linseed flax miRNAs and their targets, all of the target genes and differentially expressed targets were mapped to Gene Ontology (GO) terms(http://www.geneontology.org). The number of the genes of each term was calculated. GO terms with a p-value less than the threshold of 0.05 were considered to be significantly enriched. GO annotation results were plotted using WEGO (http://wego.genomics.org.cn/).

### 4.6. Verification of Cleavage Sites of miRNA Target Genes

In order to verify the target gene cleavage sites of miRNA, the 5’-RLM-RACE technique was applied [37]. First of all, the 500 μg total RNA by Oligolex ® mRNA mini Kit (Qiagen, Hilden, Germany) enrichment of mRNA, further use of First Choice ® RLM-RACE Kit (Ambion, Foster, CA, USA) for the enrichment of mRNA and 5’ joints, reverse transcription cDNA, the use of nested PCR for two rounds of amplification, recycling series connected to 5minTM TA/Blunt-Zero Cloning Kit (Vazyme, Nanjing, China) and sequencing. Ten to fifteen clones were used for sequencing to confirm the cleavage sites.

### 4.7. Arabidopsis Plant Transformation

In order to check whether *LumiR156a* is correlating with the agronomic traits, *miR156* over-expression vectors (Pro35S: miR156a) were constructed. To construct Pro35S: miR156a, a 0.5 KB fragment encompassing the pri-miR156a sequence was amplified (MIR156a-F: ACGGGGGACTGAATTCTGTGTAAGGACAAGAGAGGTAGC; MIR156a-R: CCGCCTCGAGCCCGGGAGTAAGGACACCTGGAGGCT) introducing *EcoR* I and *Xma* I restriction sites and subcloned into pBinGlyRed vector [38]. Plants were transformed with Agrobacterium EHA105 containing the Pro35S: *miR156*a construct. The seeds of Arabidopsis *Col*-0 ectotype were directly sown into the soil and grew in the culture room under conditions (16 h light / 8 h dark, 23 °C). Floral dip method was used to transform the wild type plants [39]. Red seeds were selected as positive under green light.

### 4.8. Phenotypes of Transgenic Arabidopsis Plant

Homozygous transgenic *Arabidopsis* lines in T3 generation contained *LumiR156a* were obtained and their characters were identified. Phenotypes of the flowering time, the number of rosette leaves and the oil content in seeds were measured. The phenotypes were collected from 12 plants in each line. Fatty acid methyl esters were extracted into hexane and analyzed by GC-MS [40]. Fatty acid methyl esters were formed by transesterification of 5–10 mg seeds by heating with MeOH-H2SO4(19:1) at 70 °C for 30 min. Fatty acid compositions were calculated against the internal control.

### 4.9. MiRNA and Gene Expression Analysis in Transgenic Arabidopsis Plants

As *SPL6* and *SPL9* genes were screened as the target genes of *Lu-miRNA156a* in linseed flax, the homologous genes of them in *Arabidopsis* genome were selected as target genes to evaluate their expression values in *Arabidopsis* transgenic lines by using qPCR method. Besides *SPL6* and *SPL9*, the gene expression values during seed development of some other SPL genes, including *SPL2*, *SPL3*, *SPL10* and *SPL11* were also evaluated. The siliques with 20DAF were used as materials. Meanwhile, the important seed oil synthesis genes, *FAD2*, *FAD3* and *FAE1* were chosen for gene expression analysis. The primers were listed in Supplementary Table S4.

## Figures and Tables

**Table 1:** The number of sequencing reads and distribution of sRNAs obtained from 4 libraries.

Samples	M5	M10	M20	M30
Raw_reads	22,179,284	34,145,577	19,947,423	18,881,760
Containing N’ reads	4476	6996	3921	3772
Length<18	432,915	20,339,545	1,489,610	1,056,229
Length>30	1,140,573	759,356	952,238	3,951,093
Clean_reads	20,601,320(100%)	13,039,680(100%)	17,501,654(100%)	13,870,666(100%)
rRNA	1,537,810(7.46%)	2,099,680(16.10%)	2,701,051(15.43%)	5,230,679(37.71%)
scRNA	0(0.00%)	0(0.00%)	0(0.00%)	0(0.00%)
snRNA	2(0.00%)	3(0.00%)	0(0.00%)	0(0.00%)
snoRNA	1364(0.01%)	5601(0.04%)	2587(0.01%)	10,255(0.07%)
tRNA	146,574(0.71%)	1,052,700(8.07%)	371,121(2.12%)	1,314,919(9.48%)
Repbase	3693(0.02%)	13,668(0.10%)	6974(0.04%)	9299(0.07%)
Unannotated	18,911,877(91.80%)	9,868,028(75.69%)	14,419,921(82.40%)	7,305,514(52.67%)
Mapped_Reads to genome	11,568,108	6,180,914	9,606,035	4,181,608

**Table 2:** Distribution of miRNAs in four libraries.

Samples	Known-miRNAs	Novel-miRNAs	Total
M5	108	117	225
M10	110	120	230
M20	104	120	224
M30	87	118	205
Total	114	121	235

**Table 3:** Fatty acids profiles of *Arabidopsis* seeds over-expressing *Lus-miR156a.*

Fatty Acids	WT	OXmiR156a-3	OXmiR156a-5	OXmiR156a-9
C16:0	0.03 ± 0.002	0.02 ± 0.003	0.021 ± 0.004 *	0.02 ± 0.004 *
C16:1^Δ9^	0.002 ± 0.001	0.002 ± 0.001	0.003 ± 0.001	0.003 ± 0.001
C18:0	0.009 ± 0.001	0.005 ± 0 **	0.006 ± 0.001 **	0.006 ± 0.001 **
C18:1^Δ9^	0.047 ± 0.006	0.033 ± 0.009	0.04 ± 0.005	0.036 ± 0.002 *
C18:2 ^Δ9,12^	0.106 ± 0.002	0.079 ± 0.013 **	0.085 ± 0.004 **	0.081 ± 0.008 **
C18:3 ^Δ9,12,15^	0.06 ± 0.005	0.042 ± 0.01 *	0.044 ± 0.005 **	0.04 ± 0.007 **
C20:0	0.004 ± 0.001	0.003±0.001	0.003 ± 0.001	0.003 ± 0.001
C20:1 ^Δ11^	0.04 ± 0.01	0.028±0.005*	0.034 ± 0.006 *	0.035 ± 0.011
C20:2 ^Δ11,14^	0.004 ± 0.001	0.003 ± 0 **	0.003 ± 0 **	0.003 ± 0 **
C22:1 ^Δ13^	0.005 ± 0.002	0.004 ± 0	0.006 ± 0.002	0.005 ± 0.002
Sum	0.323 ± 0.011	0.229 ± 0.026 **	0.25 ± 0.006 **	0.238 ± 0.024 **

* *p* < 0.05 and ** *p* < 0.01 indicated significant differences with WT.

## Supplementary Materials

Supplementary materials can be found at https://www.mdpi.com/1422-0067/21/8/2708/s1.

## Abbreviations

miRNA	microRNA
DAF	days after flowering
qPCR	quantitative real-time polymerase chain reaction
nt	nucleotide
FC	fold change
FDR	false discovery rate
TPM	transcripts per million
NGS	next-generation sequencing
GO	Gene Ontology
SPL	SQUAMOSA PROMOTER BINDING PROTEIN-LIKE
GC-MS	Gas Chromatography-Mass Spectrometer
